# Specific Direction-Based Outlier Detection Approach for GNSS Vector Networks

**DOI:** 10.3390/s19081836

**Published:** 2019-04-17

**Authors:** Yufeng Nie, Ling Yang, Yunzhong Shen

**Affiliations:** College of Surveying and Geo-informatics, Tongji University, Shanghai 200092, China; yufeng.nie@tongji.edu.cn (Y.N.); lingyang@tongji.edu.cn (L.Y.)

**Keywords:** outlier detection, GNSS networks, baseline vector, antenna height

## Abstract

In this paper we propose an outlier detection approach for GNSS vector networks based on the specific direction (i.e., SD approach), along which the test statistic constructed reaches the maximum. We derive the unit vector of this specific direction in detail, and prove that the unit vector is the same as that determined by the outlier estimates in three-dimensional (3D) approach, while the distribution of the maximum test statistic in this direction is the square root of *Chi*-squared distribution. Therefore, eliminating an outlier along this specific direction can get the same result as that of eliminating all three components of outlier vector in 3D approach. The mathematical equivalence of SD approach and 3D approach is further demonstrated by a real GNSS network. Moreover, preliminary application of the SD approach to detect the abnormal antenna height measurement is carried out in terms of numerical simulations of multiple baseline solutions, and it shows that the SD approach can effectively detect baselines that are directly infected by corresponding receiver antenna height errors.

## 1. Introduction

When a weight matrix is chosen as the inverse of observables’ covariance matrix, the weighted least-squares (WLS) estimation is the best linear unbiased estimator (BLUE), assuming that no outlier exists. However, outliers can inevitably occur in practice and cause the optimal feature of such estimation loss [1,2,3]. Therefore, outliers must be detected and then eliminated as soon as possible. Baarda [4] first introduced ‘data-snooping’ for detecting outliers in geodetic networks, where outliers are identified one by one based on the test statistic of single outlier detection approach. The test statistic can be constructed according to various statistical distributions, e.g., standard normal distribution, τ-distribution, and *F*-distribution [4,5,6,7], and one can determine the existence of outliers by a comparison with correspondent critical value at a given significance level [8,9]. Three types of errors, i.e., rejecting the right observation (type I error) and accepting the wrong observation (type II error) as well as locating the outlier to right observation (type III error) [10,11,12,13], are inevitably encountered in outlier detection processes. Therefore, the reliability theory is of fundamental importance in outlier detection, the content of which has been extended from the case for single outlier [4] to multiple outliers [7,14,15] and from independent observations to correlated ones [16,17]. According to the reliability theory, once the possibility of type I and type II errors is given, the Minimal Detectable Bias (MDB) and Bias-to-Noise Ratios (BNR), defined respectively as the measures of internal reliability and external reliability of geodetic networks, are uniquely determined [18,19,20]. Both MDB and BNR reflect the characteristics of a geodetic network to resist outliers, and the BNR shows impact of non-detected outlier on the final solution [21], which can be reduced or eliminated by robust methods via iteratively reweighting of observations [2,22,23,24,25]. The outlier detection and reliability theory has already been widely applied for the three-dimensional (3D) networks of Global Navigation Satellite System (GNSS) [26,27,28,29,30,31,32,33].

At present, outlier detection for 3D GNSS vector networks is performed either at one-dimensional (1D) baseline component level or 3D baseline vector level [34], and based on a fundamental set of independent loops [35,36] or by adding a redundancy constraint [37]. Different from many other geodetic measurements, the baseline vector observations of GNSS networks are not directly observed, but derived from the pseudo-range or carrier-phase measurements [38]. Hence, there can be various outliers’ sources in GNSS networks such as satellite orbit error, impractical tropospheric model, wrong measurement of GNSS antenna height, antenna centering and positioning, etc. These factors usually come from a specific direction in the space, and have varying effects on all coordinate components of baseline vectors [39]. Supposing a GNSS baseline vector is spoiled by the outlying antenna height measurement of a station, this outlier would be detected with a higher probability when the least-squares residuals are projected to the vertical direction of the station. Therefore, outlier detection in GNSS networks can be conducted by searching a specific direction, so that the test statistic for outliers along this direction reaches maximum. However, due to the existence of random observation errors, this specific direction is certainly deflected from the true one. How can we determine such a specific direction for outlier detection of a certain baseline vector? Further, once the outlier in this direction is detected, what is its impact on the other two coordinate components orthogonal to this specific direction? These issues motivate the following investigation of the paper.

The rest of this paper is organized as follows. Section 2 briefly reviews the traditional outlier detection approaches in GNSS vector networks and derives in detail the mathematical formulas of the SD approach. Section 3 presents the results of applying ‘data snooping’ to a real GNSS network by 1D, 3D, and the proposed Specific Direction-based (SD) approach, which validates the effectiveness of the SD approach. In Section 4, we apply the SD approach for the detection of wrong GNSS antenna height measurement in terms of numerical simulations of multiple baseline solutions. Section 5 gives the concluding remarks.

## 2. Methodology

### 2.1. Traditional Outlier Detection Approach for GNSS Vector Observations

Since it is usually impossible to predetermine the number of outliers, hypothesis testing is practically applied by iteratively removing the wrong observation corresponding to the largest test statistic until no statistic exceeds the critical value. If a single outlier $\nabla_{i}$ occurs at the *i*th observation, the general linear or linearized observation equation is expanded to

(1) $$y=Ax+h_{i}\nabla_{i}+\varepsilon$$

where y is a 3*m* × 1 vector of observations with *m* the number of baselines, A is a 3*m* × *n* design matrix with full column rank, x is an *n* × 1 vector of unknown parameters to be solved, $h_{i}={\left(\begin{array}{ccccccc} 0 & ï & 0 & 1 & 0 & ï & 0 \end{array}\right)}^{T}$ is a 3*m*-dimensional zero vector with only the *i*th element equaling to one and ε is an 3*m* × 1 vector of observation error with the covariance Σ. When the weight matrix in WLS is taken as $P=\sigma_{0}^{2}\Sigma^{-1}$ with $\sigma_{0}^{2}$ the prior variance factor, the least-squares estimate of the outlier ${\hat{\nabla }}_{i}$ and its variance $\sigma_{{\hat{\nabla }}_{i}}^{2}$ are as follows

(2) $${\hat{\nabla }}_{i}={\left(h_{i}^{T}PQ_{vv}Ph_{i}\right)}^{-1}h_{i}^{T}PQ_{vv}Py={\left(h_{i}^{T}\bar{P}h_{i}\right)}^{-1}h_{i}^{T}\bar{P}y,\;\sigma_{{\hat{\nabla }}_{i}}^{2}=\sigma_{0}^{2}{\left(h_{i}^{T}\bar{P}h_{i}\right)}^{-1}$$

where $\bar{P}=PQ_{vv}P$ is called the reliability matrix of the observations [20], and $Q_{vv}=P^{-1}-A{\left(A^{T}PA\right)}^{-1}A^{T}$ is the cofactor matrix of residuals vector $v=-Q_{vv}Py$. Then the *w*-test statistic for the *i*th observation is formulated as [4,7]:

(3) $$w_{i}=\frac{{\hat{\nabla }}_{i}}{\sigma_{{\hat{\nabla }}_{i}}}=\frac{h_{i}^{T}\bar{P}y}{\sigma_{0}\sqrt{\left(h_{i}^{T}\bar{P}h_{i}\right)}}$$

If there is no outlier in the *i*th observation, $w_{i}$ follows the standard normal distribution, i.e., $w_{i}∼N\left(0,1\right)$. Once the significance level $\alpha_{0}$ of the test is given, the critical value of test statistic is uniquely determined by the distribution function. If the absolute value of $w_{i}$ is larger than the critical value, the estimated outlier ${\hat{\nabla }}_{i}$ by Equation (2) is assumed significant and thereby an outlier is detected in the *i*th observation. If the variance factor $\sigma_{0}^{2}$ in (3) is unknown and substituted with its unbiased estimator ${\hat{\sigma }}_{0}^{2}$, then the test statistic (3) follows the τ-distribution with *r* − 1 degrees of freedom with *r* being the redundancy number of observations [5,6]. In particular, for uncorrelated observations case, test statistic (3) can be simplified as

(4) $$w_{i}=\frac{v_{i}}{\sigma_{0}\sqrt{q_{v_{i}v_{i}}}}$$

where $v_{i}$ and $q_{v_{i}v_{i}}$ denote the *i*th element of the residuals vector v and the *i*th diagonal element of cofactor matrix $Q_{vv}$ in (2), respectively.

The Minimal Detectable Bias (MDB), as a measure of internal reliability, of the *i*th observation for single outlier case is expressed as [4,7]:

(5) $$\mathrm{MDB}\left(\nabla_{i}\right)=\frac{\delta_{0}\sigma_{0}}{\sqrt{h_{i}^{T}\bar{P}h_{i}}}$$

where $\delta_{0}$ is the non-centrality parameter, which is uniquely determined by the size of type I error $\alpha_{0}$ and type II error $\beta_{0}$ [40]. 

For the GNSS baseline networks, it is reasonable to treat the baseline vector observations in triples manner because the three components of a baseline vector are computed together by the same GNSS observations and are naturally correlated. Once an outlier occurs, all three components would be impacted. Therefore, the 3D outlier detection approach is intuitively developed specifically for the GNSS baseline vector applications [34]. To describe the 3D approach, the observation vector and design matrix in (1) is partitioned as

(6) $$y=\left(\begin{array}{c} y_{1}\\ y_{2}\\ \vdots \\ y_{m} \end{array}\right),\;A=\left(\begin{array}{c} A_{1}\\ A_{2}\\ \vdots \\ A_{m} \end{array}\right),\;\varepsilon =\left(\begin{array}{c} \varepsilon_{1}\\ \varepsilon_{2}\\ \vdots \\ \varepsilon_{m} \end{array}\right)$$

where $y_{j}$ and $\varepsilon_{j}$ are the 3 × 1 vectors of the *j*th baseline observation and observation error, $A_{j}$ is the *j*th 3 × *n* design sub-matrix. When the *i*th observation vector $y_{i}$ contains a 3D outlier vector $d_{i}$, Equation (1) is rewritten with (6) as

(7) $$y_{j}=A_{j}x+H_{j}d_{i}+\varepsilon_{j},\;j=1,2,\ldots ,m$$

and $H_{j}=\{\begin{array}{c} 0_{3},\;\mathrm{for}\;j\neq i\\ I_{3}\;\mathrm{for}\;j=i \end{array}$, where $0_{3}$ is a 3 × 3 zero matrix and $I_{3}$ is a 3 × 3 identity matrix. The WLS estimation ${\hat{d}}_{i}$ of 3D outlier vector is derived from (7) as

(8) $${\hat{d}}_{i}={\bar{P}}_{ii}^{-1}\sum_{j=1}^{m}{\bar{P}}_{ij}y_{j}$$

in which, ${\bar{P}}_{ij}$ denotes the *ij*-th 3 × 3 sub-matrix of the reliability matrix $\bar{P}$. To determine whether or not the 3D vector of outliers exists, the test statistic $T_{i}$ is constructed by

(9) $$T_{i}=\frac{{\hat{d}}_{i}^{T}{\bar{P}}_{ii}{\hat{d}}_{i}/3}{\sigma_{0}^{2}}$$

If there are no outliers, $T_{i}$ is central *F*-distributed with two degrees of freedom as 3 and ∞ at given $\alpha_{0}$, i.e., $T_{i}∼F(\alpha_{0};3,\infty )$. If the variance factor $\sigma_{0}^{2}$ in (9) is unknown, then $T_{i}$ can be re-constructed following the central *F*-distribution of $F\left(\alpha_{0};3,3m-n-3\right)$ if no outlier exists according to [6] (p. 302).

### 2.2. Specific Direction-Based (SD) Approach for GNSS Vector Observations

#### 2.2.1. Outlier Detection in SD Approach

Supposing the outlier’s coefficient matrix of (7) is defined as $H_{j}=\{\begin{array}{c} 0_{3},\;\mathrm{for}\;j\neq i\\ u_{ik}\;\mathrm{for}\;j=i \end{array}$, where $u_{ik}$ represents the 3D directional cosines relative to three Cartesian coordinate axes and $0_{3}$ is a 3D zero vector, similar to (3) the test statistic for the *i*th baseline vector observation at the *k*th direction of is constructed as

(10) $$w_{ik}=\frac{u_{ik}^{T}\sum_{j=1}^{m}{\bar{P}}_{ij}y_{j}}{\sigma_{0}\sqrt{u_{ik}^{T}{\bar{P}}_{ii}u_{ik}}}=\frac{u_{ik}^{T}g_{i}}{\sigma_{0}\sqrt{u_{ik}^{T}{\bar{P}}_{ii}u_{ik}}}=\frac{g_{i}^{T}u_{ik}}{\sigma_{0}\sqrt{u_{ik}^{T}{\bar{P}}_{ii}u_{ik}}}$$

where $g_{i}=\sum_{j=1}^{m}{\bar{P}}_{ij}y_{j}$ is a 3D vector and ${\bar{P}}_{ij}$ is the *ij*-th 3 × 3 sub-matrix of the reliability matrix $\bar{P}$. For different directions $u_{ik}$, the testing values $w_{ik}$ in (10) are also different. 

Accordingly, the outlier detection and identification should focus on finding the specific unit direction vector, supposing $u_{i3}$, that enables the largest test statistic for (10). This can be solved by the following target function $\Phi \left(u_{i3}\right)$

(11) $$\max:\Phi \left(u_{i3}\right)=w_{i3}^{2}=\frac{{\left(g_{i}^{T}u_{i3}\right)}^{2}}{\sigma_{0}^{2}u_{i3}^{T}{\bar{P}}_{ii}u_{i3}}$$

For a local maximum of the target function above, its first order partial derivative must equal to zero, i.e.,

(12) $$\frac{\partial \Phi \left(u_{i3}\right)}{\partial u_{i3}}=\frac{2\left(g_{i}^{T}u_{i3}\right)\left[\left(u_{i3}^{T}{\bar{P}}_{ii}u_{i3}\right)g_{i}^{T}-\left(g_{i}^{T}u_{i3}\right)u_{i3}^{T}{\bar{P}}_{ii}\right]}{\sigma_{0}^{2}{\left(u_{i3}^{T}{\bar{P}}_{ii}u_{i3}\right)}^{2}}=0$$

The solution of (12) is

(13) $$u_{i3}=\frac{u_{i3}^{T}{\bar{P}}_{ii}u_{i3}}{g_{i}^{T}u_{i3}}{\bar{P}}_{ii}^{-1}g_{i}\;\mathrm{and}\;g_{i}^{T}u_{i3}=0$$

where the matrix ${\bar{P}}_{ii}^{-1}$ denotes the inverse of ${\bar{P}}_{ii}$. When $g_{i}^{T}u_{i3}=0$, the test statistic of (10) gets the minimum value, which is not the right solution we are looking for. Since the scalar factor $u_{i3}^{T}{\bar{P}}_{ii}u_{i3}/g_{i}^{T}u_{i3}$ in (13) does not impact the direction of the unit direction vector $u_{i3}$, the first equation of (13) is simply equivalent to (14) for determination of a spatial direction

(14) $$u_{i3}=\pm \frac{{\bar{P}}_{ii}^{-1}g_{i}}{\left\|{\bar{P}}_{ii}^{-1}g_{i}\right\|}$$

with $\left\|{\bar{P}}_{ii}^{-1}g_{i}\right\|=\sqrt{g_{i}^{T}{\bar{P}}_{ii}^{-2}g_{i}}$ being the 2-norm of the vector ${\bar{P}}_{ii}^{-1}g_{i}$. Here, by comparing (14) with (8), we can find that $u_{i3}$ and ${\hat{d}}_{i}$ are along the same direction, indicating the outlier vector derived from 3D approach can intrinsically determine the specific direction with maximum test statistic. 

By taking the second order partial derivative to (12) and then substituting (14) into it, one can get

(15) $$\frac{\partial \Phi^{2}\left(u_{i3}\right)}{\partial u_{i3}^{2}}=-\frac{{\bar{P}}_{ii}\left[\left(g_{i}^{T}{\bar{P}}_{ii}^{-1}g_{i}\right)I_{3}-{\bar{P}}_{ii}^{-1}g_{i}g_{i}^{T}\right]}{\sigma_{0}^{2}g_{i}^{T}{\bar{P}}_{ii}^{-1}g_{i}}$$

where $I_{3}$ denotes the 3 × 3 identity matrix. Since the block matrix ${\bar{P}}_{ii}$ is positive definite, if the matrix $M_{i}=\left(g_{i}^{T}{\bar{P}}_{ii}^{-1}g_{i}\right)I_{3}-{\bar{P}}_{ii}^{-1}g_{i}g_{i}^{T}$ is non-negative definite, the solution of (14) is the unique unit vector to get the local maximum test statistic, and it must be the global maximum one. By substituting (14) into (10), one can derive the maximum value as (16), which is utilized as test statistic in the SD approach

(16) $$\left|w_{i3}\right|=\frac{1}{\sigma_{0}}\sqrt{g_{i}^{T}{\bar{P}}_{ii}^{-1}g_{i}}$$

Considering $g_{i}=\sum_{j=1}^{m}{\bar{P}}_{ij}y_{j}$, test statistic (16) can be rewritten with (8) as

(17) $$\left|w_{i3}\right|=\frac{1}{\sigma_{0}}\sqrt{{\left({\bar{P}}_{ii}^{-1}g_{i}\right)}^{T}{\bar{P}}_{ii}{\bar{P}}_{ii}^{-1}g_{i}}=\frac{1}{\sigma_{0}}\sqrt{{\hat{d}}_{i}^{T}{\bar{P}}_{ii}{\hat{d}}_{i}}$$

Comparing (17) with (9), we can find that ${\left|w_{i3}\right|}^{2}$ follows the *Chi*-squared distribution with 3 degrees of freedom. Thereby, the SD approach is mathematically equivalent to the 3D method and its critical value for $\left|w_{i3}\right|$ can be directly calculated by $\sqrt{3\cdot F\left(\alpha_{0};3,\infty \right)}$.

#### 2.2.2. Outlier Elimination in SD Approach

If the maximum test statistic $\left|w_{i3}\right|$ by (17) is larger than its critical value, the 3D test statistic (9) will also be larger than its corresponding critical value and the whole *i*th baseline vector should be eliminated. 

Evaluating 3D outlier estimates (8), it can be rewritten as

(18) $${\hat{d}}_{i}={\bar{P}}_{ii}^{-1}\sum_{j=1}^{m}{\bar{P}}_{ij}y_{j}={\bar{P}}_{ii}^{-1}g_{i}=\frac{{\bar{P}}_{ii}^{-1}g_{i}}{\left\|{\bar{P}}_{ii}^{-1}g_{i}\right\|}\left\|{\bar{P}}_{ii}^{-1}g_{i}\right\|=u_{i3}d_{i3}$$

where $d_{i3}=\left\|{\bar{P}}_{ii}^{-1}g_{i}\right\|$, the outlier estimates at the other two directions ($u_{i1}$ and $u_{i2}$) orthogonal to $u_{i3}$ must be zero. Therefore, in SD approach, if an outlier occurs at the *i*th baseline vector, the observational equation for eliminating the outlier along the specific direction $u_{i3}$ is expressed as

(19) $$\begin{array}{l} y_{i}=A_{i}x+u_{i3}d_{i3}+\varepsilon_{i},\;\mathrm{and}\\ y_{j}=A_{j}x+\varepsilon_{j},\;j=1,2,\ldots ,m;j\neq i \end{array}$$

Then the estimates of parameter vector x and its variance can be derived via least squares adjustment. It was found that elimination of the outlier estimated in this direction will lead to the same results of elimination outlier vector ${\hat{d}}_{i}$ in the whole baseline vector as done in 3D approach, since the outlier scalar estimated in this specific direction $u_{i3}$ contains all the information content of 3D outlier vector ${\hat{d}}_{i}$.

## 3. Outlier Detection and Elimination for Real GNSS Network

### 3.1. Data Description

**Figure 1:**
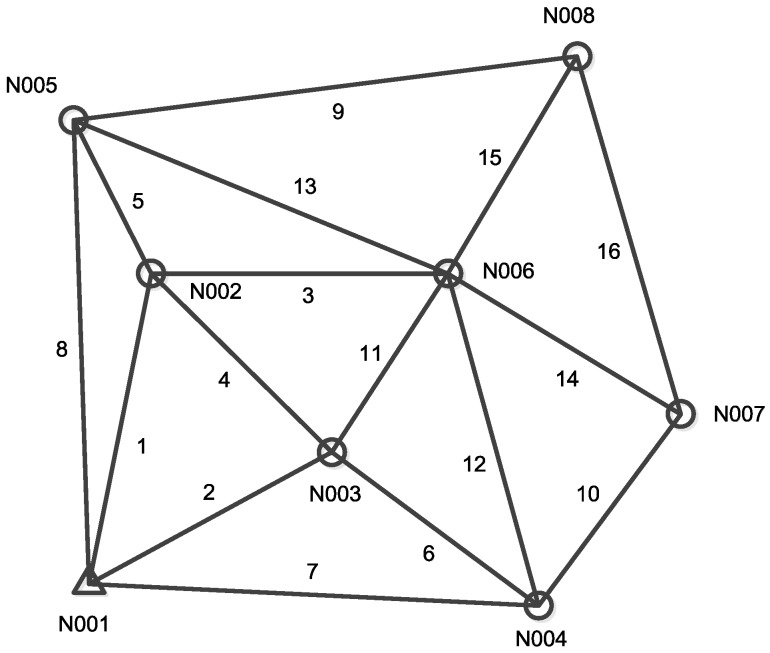
Shape of the GNSS network.

The real GNSS network used in the following is shown in Figure 1 and its observation data set is given in Table A1 and Table A2 in the Appendix [41]. There are 8 sites and 16 baselines in this network, and the site N001 is fixed as known for the free network adjustment.

The square root of prior variance factor $\sigma_{0}$ is taken as 1 cm in the following data analysis, and probabilities of type I and type II errors are chosen as $\alpha_{0}=0.1\%$ and $\beta_{0}=20\%$ respectively thereafter [7].

### 3.2. Specific Direction Validation

**Figure 2:**
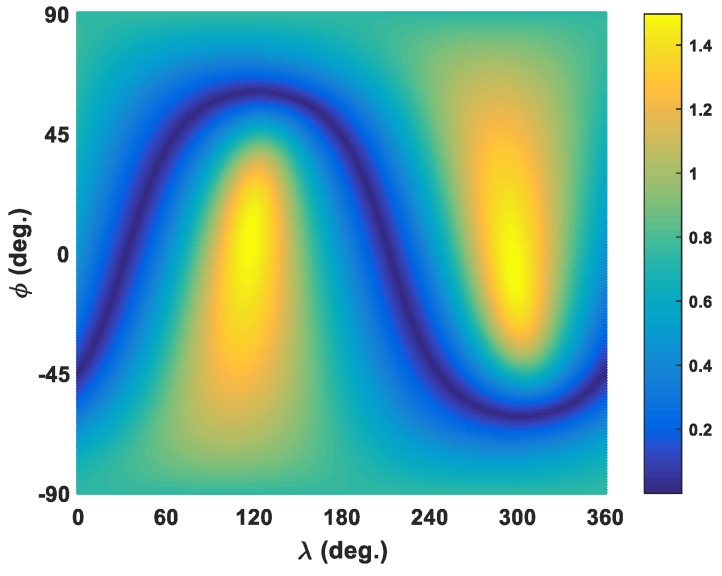
The absolute test statistic values of the No. 1 baseline by numerical traversal algorithm.

**Figure 3:**
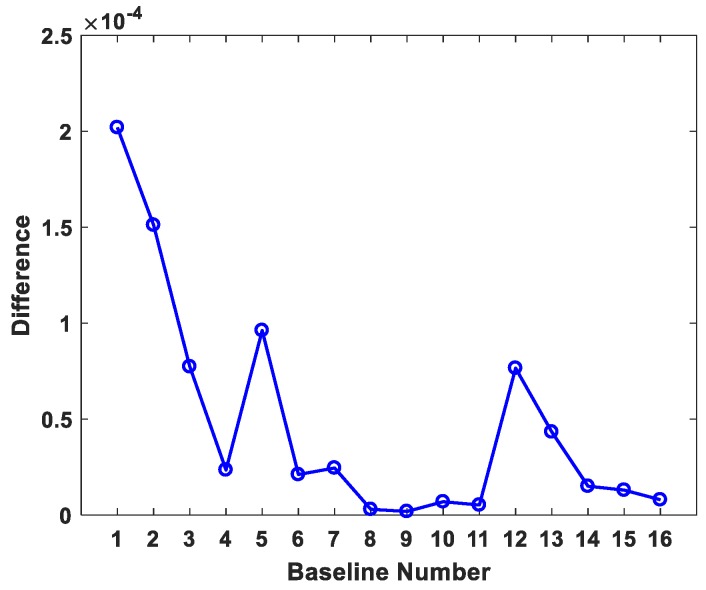
Differences between maximum absolute statistics by two calculation approaches.

If the matrix $M_{i}=\left(g_{i}^{T}{\bar{P}}_{ii}^{-1}g_{i}\right)I_{3}-{\bar{P}}_{ii}^{-1}g_{i}g_{i}^{T}$ in (15) is non-negative definite, previous derivation guarantees the unit direction vector $u_{i3}$ determined by (14) is the specific direction for the *i*th baseline observation to achieve the maximum test statistic as expressed by (16). The unit direction vector $u_{ik}$ of an arbitrary direction in 3D space can be expressed as

(20) $$u_{ik}={\left(\begin{array}{ccc} \cos\phi \cos\lambda & \cos\phi \sin\lambda & \sin\phi \end{array}\right)}^{T}$$

where $\phi \in [-90^{\circ },90^{\circ }]$ and $\lambda \in [0^{\circ },360^{\circ }]$ are spherical coordinates. When ϕ and λ are fixed, correspondent test statistic of (10) at this direction is uniquely determined. Therefore, the specific direction, which (10) generates as the maximum test statistic, can be obtained by simply traversing the whole range space of ϕ and λ given a certain small step size. To validate the effectiveness of (14) and (16), statistic values (10) of the No. 1 baseline for all (ϕ,λ) direction combinations are calculated and plotted in Figure 2 given the $1^{\circ }$ step size. It is shown that values in Figure 2 manifest a symmetric pattern with respect to the origin and there exist two maximum points of 1.4973 in two opposite directions, just corresponding to the positive and negative sign in (14). This value is slightly smaller than the maximum 1.4975, which is directly derived from analytical formula (16). The differences of the maximum test statistics by analytical formula (16) proposed in SD method and those by numerical traversal algorithm are shown in Figure 3 for 16 baseline observations, which indicate that the maximum test statistics derived from (16) are all slightly larger than those from the traversal method. It further proves the test statistic derived from (16) is the theoretical global maximum one, since the traversal method can only get an approximate maximum due to the step size limitation. Besides this, the matrices $M_{i}=\left(g_{i}^{T}{\bar{P}}_{ii}^{-1}g_{i}\right)I_{3}-{\bar{P}}_{ii}^{-1}g_{i}g_{i}^{T}$ corresponding to 16 baseline observations are all non-negative definite.

### 3.3. Outlier Detection

The test statistics of all baseline observations of the networks by SD, 3D, and 1D approach, calculated via (16), (9), and (3) respectively, are listed in Table 1, and the correspondent spherical coordinates denoting the specific directions in SD approach are also demonstrated. For a given $\alpha_{0}=0.1\%$, the critical values of three approaches are shown in Table 2, which are respectively computed by the inverse of the standard normal cumulative distribution function $N\left(\alpha_{0};0,1\right)$ for the 1D approach, by inverse of the central cumulative *F*-distribution function $F\left(\alpha_{0};3,\infty \right)$ for 3D approach, and by $\sqrt{3\cdot F\left(\alpha_{0};3,\infty \right)}$ for SD approach. Besides this, test statistics larger than corresponding critical values are marked in bold font.

Since one outlier can pollute its neighboring observations and possibly causes their test statistic values exceed the critical value, the ‘data snooping’ procedure is iteratively conducted, i.e., detecting the outliers one by one. The largest test statistics among all baseline observations in each test step by different approaches are shown in Table 3 and those exceeding critical values are in bold font.

In Step 1, the outlier is detected at the No. 3 baseline by all three approaches, but the 1D method locates the outlier only at the *Y*-component of the baseline. Therefore, the No. 3 baseline observation should be discarded and the remaining data set is used to continue ‘data snooping’ procedure in Step 2. In Step 2, all test statistics are well lower than the corresponding critical values listed in Table 2; therefore, no outlier is detected, indicating that the current dataset is quite ‘clean’ and the ‘data snooping’ procedure can be terminated. However, three methods show obvious discrepancy at this step. By the SD and 3D method, the largest statistics are reached both at the No. 1 baseline. However, by the 1D method, the largest test statistic is located at the *Z*-component of the No. 9 baseline, indicating that the baseline-component-based method (1D approach) and the baseline-vector-based method (3D and SD approach) do not always lead to same results as already discussed by [34].

### 3.4. Outlier Elimination

**Figure 4:**
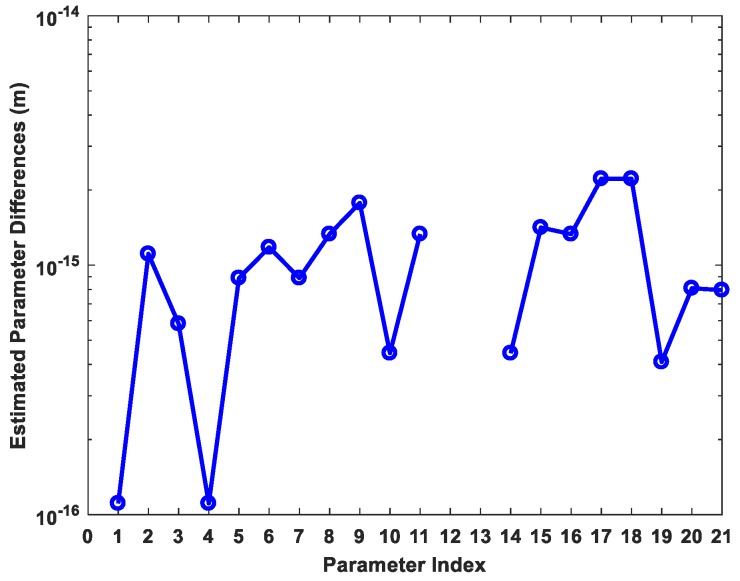
The absolute value of differences between SD and 3D approach derived parameter estimation after outlier elimination. The values of parameter 12 and 13 are zero due to floating-point number round-off.

After identifying the outlier observation, its influence on the final parameter estimation must be eliminated. Since the SD approach is mathematically equivalent to the 3D approach, it can be expected that eliminating the influence of outlier in specific direction (14) has the same effect as that in 3D approach by (8). Figure 4 shows the absolute differences of 21 coordinate parameters (7 unknown sites) estimated by SD and 3D approach after elimination of outliers’ influence respectively. The differences are ignorable and are merely caused by the limits of computer precision. Note that the values of parameters 12 and 13 are zero due to floating point number round-off and therefore not be presented in Figure 4. The final parameters estimation after outlier elimination of the No. 3 baseline by SD and 3D method are listed in Table 4.

## 4. Simulation Analysis for Detecting Abnormal GNSS Antenna Height Measurements

In this section, we adopt the SD approach to detect baseline vectors which are infected by abnormal antenna height measurement in the GNSS networks in terms of numerical simulations of multiple baseline solutions. The GNSS network used for simulations is based on Figure 1, which also consists of 8 sites and 16 baselines. Simulated baseline vector observations are generated in two steps, where firstly error-free baseline vectors are calculated by ‘true’ coordinates of each site, and secondly baseline vector noises are randomly generated according to corresponding baseline covariance matrix and then added to the error-free baseline vector. The simulation procedure is described in detail by [23], and in following simulations the estimated sites coordinates in Table 4, as well as the known coordinates of site N001 in Appendix A, are treated as the ‘true’ values for error-free baseline vector generation. Besides this, the covariance matrices in Appendix A are used to generate baseline vector noises as that in [23].

Assuming there are four GNSS receivers to carry out the measurement task of above-mentioned GNSS network, the surveying is divided into six observation sessions as arranged in Table 5 and note that only three receivers are used for sessions 2 and 5. In each session, there are at most three functional independent baseline vectors for the final network adjustment. Since the multiple baseline solutions are supposed, the baselines of each session must be stochastic dependent and the correlation coefficients from 0.2 to 0.3 between different baselines’ components are assumed during the construction of weight matrix. In the following simulations, it is assumed that the GNSS antenna height on site N006 is wrongly measured by 10 cm in observation session 2, which is possibly caused by, for example, the misreading of antenna height. Therefore, baselines No. 3 and No. 11 are directly influenced by the wrong N006 antenna height. The influence is introduced by upward continuation of the site N006 coordinates in the error-free baseline vector generation step of observation session 2, while for other sessions the coordinates of site N006 are still based on that in Table 4. 

The numerical simulations are carried out 10,000 times, and for each simulation the baseline vector noises are newly produced while the 10 cm antenna height error of site N006 in session 2 is kept fixed. We use the SD approach to estimate the spatial direction, i.e., latitude and longitude, of possible outlier vector for each baseline by (14), and calculate the standard deviation (SD) of them with respect to the upward direction of site N006 by (21). In Equation (21), $SD_{\varphi }$ and $SD_{\lambda }$ stand for the SD values of latitude and longitude estimates respectively, and the upward direction of site N006 is $\left(\varphi_{N006},\lambda_{N006})=(31.3^{\circ },121.3^{\circ }\right)$ according to Table 4.

(21) $$SD_{\varphi }=\sqrt{\frac{\sum_{i=1}^{N}{\left(\varphi_{i}-\varphi_{N006}\right)}^{2}}{N}},\;SD_{\lambda }=\sqrt{\frac{\sum_{i=1}^{N}{\left(\lambda_{i}-\lambda_{N006}\right)}^{2}}{N}}\;(N=10,000)$$

**Figure 5:**
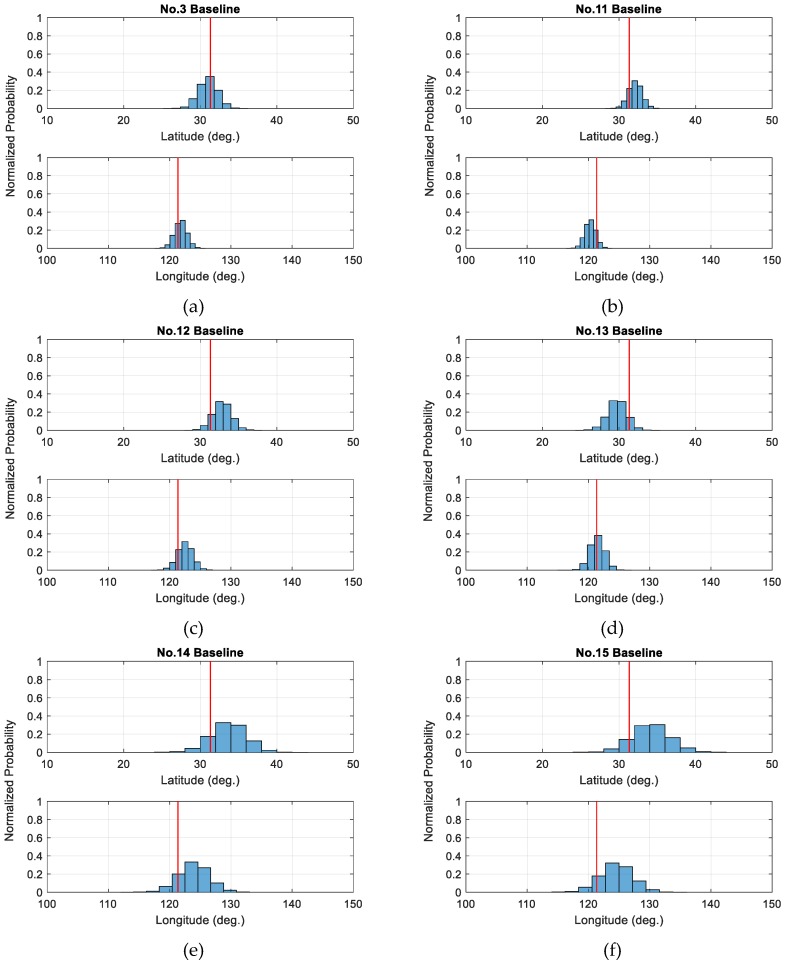
Statistical distribution of outlier direction estimates for site N006 related baselines. (**a**) for No.3 baseline, (**b**) for No.11 baseline, (**c**) for No.12 baseline, (**d**) for No.13 baseline, (**e**) for No.14 baseline and (**f**) for No.15 baseline (The red vertical lines stand for the latitude and longitude of site N006’s antenna height direction.).

Table 6 lists the SD values of both latitude and longitude estimates for all baselines as well as corresponding mean values over 10,000 simulations. As shown, the No.3 baseline reaches the best consistency in terms of outlier direction estimation with site N006’s antenna height direction, which is followed by the No.11 baseline for its smaller SD values compared to remaining baselines. Since the No. 3 and No. 11 are directly infected by the wrong antenna height of site N006 in session 2, the statistical results demonstrate that the SD approach can effectively determine the influence of wrong antenna height on baseline vectors in the GNSS network. Furthermore, we investigate the outlier direction estimates of other site N006-related baselines, which are observed in other sessions where the antenna height is correctly measured. Figure 5 shows the statistical distribution of outlier direction estimates for these baselines, from which we can obviously see more gathering outlier direction estimates for No. 3 and No. 11 baselines and smaller bias with respect to the antenna height direction of site N006 indicated by the corresponding red vertical line at each panel. Wih regard to the test statistics for each baseline observation calculated by (16), it turns out that among 16 baselines over 10,000 simulations, the No. 11 baseline reaches the maximum at about 99% times while the remaining part of the maximum values falls into the No. 3 baseline, which are all well beyond the critical value listed in Table 2. Therefore, it is possible to apply the SD method to detect the influence of wrongly measured receiver antenna height on baseline vectors in GNSS networks, which needs further investigation.

## 5. Conclusions

In this contribution, we proposed the specific direction-based outlier detection approach (SD approach), for 3D GNSS networks. By seeking the specific direction in the 3D space, the maximum test statistic of baseline vector observations is constructed. The analytical expression (14) is derived to directly obtain this specific direction and to construct the corresponding test statistic by (16). Compared to traditional 3D approach, the SD approach is derived from another point of view. It tests the baseline vector in a specific direction in which the outlier vector manifests the largest test statistic value. Evaluating (17) and (9) demonstrates that the two approaches are rigorously mathematically equivalent, while if readers want to directly investigate the spatial direction characteristic of outlier sources in the GNSS networks, the SD approach is preferred. A real GNSS network is processed to validate the effectiveness of the SD approach and the equivalence to the 3D method. Moreover, preliminary application of SD approach to detect the influence of wrong GNSS antenna height measurement on baseline vectors in the GNSS networks are carried out, which shows promising results and needs further investigation.

## Figures and Tables

**Table 1:** Test Statistics of baseline observations by Specific Direction-based (SD), 3D, and 1D approach.

Baseline Num.	SD Approach			3D Approach	1D Approach		
	λ (Deg.)	ϕ (Deg.)	Test Statistics	Test Statistics	X	Y	Z
1	5.8	118.5	1.498	0.748	0.469	1.031	0.743
2	−17.7	307.7	1.730	0.997	0.908	0.742	0.518
3	52.7	210.0	**4.378**	**6.388**	2.395	**3.469**	2.305
4	3.2	268.1	2.316	1.788	1.262	2.313	0.699
5	34.7	267.7	2.982	2.964	0.937	2.568	2.162
6	27.2	156.2	1.604	0.858	1.422	0.670	0.287
7	61.5	327.9	1.768	1.042	0.866	0.278	1.647
8	−34.2	148.0	1.993	1.324	1.425	0.101	1.527
9	83.0	213.3	2.685	2.403	0.151	1.229	2.648
10	−63.4	130.8	1.000	0.333	0.375	0.496	0.975
11	18.0	63.6	0.712	0.169	0.608	0.588	0.083
12	−19.3	344.5	2.014	1.352	1.939	0.847	0.203
13	0.3	118.2	1.542	0.792	0.308	1.184	0.990
14	−5.7	315.9	0.543	0.098	0.349	0.217	0.339
15	70.2	141.1	1.931	1.243	0.127	0.788	1.854
16	66.8	140.2	0.736	0.180	0.021	0.299	0.693

**Table 2:** Critical values of SD, 3D, and 1D test statistics.

	SD Approach	3D Approach	1D Approach
**Critical Values**	4.033	5.422	3.291

**Table 3:** Largest test statistics in each outlier detection step.

Test Step	Baseline Num.	SD Approach	3D Approach	1D Approach		
				X	Y	Z
1	3	**4.378**	**6.388**	2.395	**3.469**	2.305
2	1	2.413	1.941	0.101	2.154	1.108
	9	2.307	1.774	0.656	0.702	2.301

**Table 4:** Final sites’ coordinate parameters estimation after elimination of outliers determined by SD or 3D method.

Site	X (m)	Y (m)	Z (m)
N002	−2830634.7415	4649557.6508	3313013.3273
N003	−2831170.1981	4649484.1775	3312659.4277
N004	−2831820.5247	4649349.1169	3312296.9359
N005	−2830250.6519	4649506.9814	3313403.5257
N006	−2831231.1017	4649166.3913	3313046.1881
N007	−2832003.8156	4648890.1430	3312775.1533
N008	−2831387.7285	4648523.2569	3313809.5058

**Table 5:** Session arrangement for the observation of 16 baselines in the network by 4 GNSS receivers.

Session	Receiver Station	Baseline No.
1	N001, N002, N003, N005	1, 2, 8
2	N002, N003, N006	3, 11
3	N002, N003, N005, N006	4, 5, 13
4	N005, N006, N007, N008	9, 15, 16
5	N004, N006, N007	10, 14
6	N001, N003, N004, N006	6, 7, 12

**Table 6:** Mean and SD values of outlier direction (latitude and longitude) estimates for all baselines (unit: degree).

Baseline No.	Mean Lat.	Mean Long.	SD Lat.	SD Long.
1	22.7	117.7	10.3	7.7
2	25.9	114.1	7.6	8.5
**3**	**31.0**	**121.8**	**1.2**	**1.1**
4	15.9	97.2	18.5	70.9
5	25.1	118.8	6.5	3.0
6	26.1	118.7	5.3	2.8
7	15.8	113.6	16.0	8.9
8	17.7	115.4	14.1	6.6
9	31.7	123.1	2.5	3.0
10	31.7	122.4	2.0	2.3
**11**	**32.1**	**120.3**	**1.2**	**1.4**
12	32.8	122.5	1.9	1.7
13	29.7	121.4	2.0	1.2
14	33.6	123.9	3.2	3.5
15	34.2	124.7	3.8	4.3
16	26.1	129.7	13.2	49.7

## Appendix A

The baseline vector observations and their covariance matrix of real GNSS networks are listed in Table A1 referred to in [41], where the accurate known site N001’s 3D coordinates are: X=−2830754.6300, Y=4650074.3450, and Z=3312175.0540, with the unit of meter. The approximate 3D coordinates of seven unknown sites are listed in Table A2.

**Table A1:** Baseline vector observations and covariance matrix.

Bl.Num	Sta.Po	End.Po	ΔX (m)	ΔY (m)	ΔZ (m)	Covariance Matrix ($\times 10^{-6}$)		
1	N002	N001				1.5616		
			−119.8880	516.6920	−838.2730	−1.2684	2.5332	
						−1.6092	1.6192	3.5764
2	N003	N001	415.5670	590.1690	−484.3730	0.9704		
						−0.7912	1.5756	
						−0.9936	1.0044	2.2228
3	N006	N002	596.3630	391.2610	−32.8650	0.8868		
						−0.7200	1.5160	
						−0.8576	0.9132	1.9000
4	N002	N003	−535.4570	−73.4720	−353.8990	0.9180		
						−0.8868	2.1596	
						−0.2988	0.5916	0.9604
5	N002	N005	384.0890	−50.6680	390.1980	1.0084		
						−0.9792	2.3960	
						−0.3232	0.6472	1.0364
6	N003	N004	−650.3260	−135.0610	−362.4920	0.6004		
						−0.5796	1.3952	
						−0.1932	0.3832	0.6248
7	N004	N001	1065.8940	725.2290	−121.8830	1.1984		
						−1.1608	2.7032	
						−0.3748	0.7292	1.1796
8	N005	N001	−503.9770	567.3630	−1228.4710	1.0196		
						−0.9888	2.3016	
						−0.3172	0.6192	1.0156
9	N005	N008	−1137.0770	−983.7240	405.9790	0.8212		
						−0.8012	1.9432	
						−0.2528	0.5148	0.8316
10	N004	N007	−183.2910	−458.9740	478.2180	1.0352		
						−0.8360	1.4972	
						−0.7420	0.9900	1.3932
11	N006	N003	60.9040	317.7860	−386.7610	0.9424		
						−0.7692	1.3572	
						−0.6940	0.9112	1.2836
12	N006	N004	−589.4240	182.7260	−749.2520	1.1232		
						−0.9152	1.5996	
						−0.8368	1.0832	1.5328
13	N006	N005	980.4510	340.5890	357.3370	1.3940		
						−1.1380	1.9908	
						−1.0396	1.3508	1.9056
14	N006	N007	−772.7140	−276.2480	−271.0350	1.3328		
						−1.0844	1.8956	
						−0.9880	1.2792	1.8108
15	N008	N006	156.6270	643.1340	−763.3190	1.2804		
						−1.0448	1.8336	
						−0.9552	1.2444	1.7568
16	N008	N007	−616.0870	366.8860	−1034.3530	1.4576		
						−1.1908	2.1180	
						−1.0624	1.4064	1.9852

**Table A2:** Approximate coordinates of the solved-for sites.

Site	*X* (m)	*Y* (m)	*Z* (m)
N002	−2830634.7412	4649557.6514	3313013.3268
N003	−2831170.1980	4649484.1773	3312659.4277
N004	−2831820.5247	4649349.1166	3312296.9360
N005	−2830250.6519	4649506.9812	3313403.5257
N006	−2831231.1022	4649166.3910	3313046.1886
N007	−2832003.8159	4648890.1427	3312775.1536
N008	−2831387.7286	4648523.2565	3313809.5059
